# Striated muscle gene therapy for the treatment of lipoprotein lipase deficiency

**DOI:** 10.1371/journal.pone.0190963

**Published:** 2018-01-05

**Authors:** Katherine E. Gadek, Hong Wang, Monica N. Hall, Mitchell Sungello, Andrew Libby, Drew MacLaskey, Robert H. Eckel, Bradley B. Olwin

**Affiliations:** 1 Department of Molecular, Cellular and Developmental Biology, University of Colorado Boulder, Boulder, Colorado United States of America; 2 Division of Endocrinology, Metabolism, and Diabetes, University of Colorado Denver Anschutz Medical Campus, Aurora, Colorado United States of America; Rutgers University Newark, UNITED STATES

## Abstract

Excessive circulating triglycerides due to reduction or loss of lipoprotein lipase activity contribute to hypertriglyceridemia and increased risk for pancreatitis. The only gene therapy treatment for lipoprotein lipase deficiency decreases pancreatitis but minimally reduces hypertriglyceridemia. Synthesized in multiple tissues including striated muscle and adipose tissue, lipoprotein lipase is trafficked to blood vessel endothelial cells where it is anchored at the plasma membrane and hydrolyzes triglycerides into free fatty acids. We conditionally knocked out lipoprotein lipase in differentiated striated muscle tissue lowering striated muscle lipoprotein lipase activity causing hypertriglyceridemia. We then crossed lipoprotein lipase striated muscle knockout mice with mice possessing a conditional avian retroviral receptor gene and injected mice with either a human lipoprotein lipase retrovirus or an mCherry control retrovirus. Post-heparin plasma lipoprotein lipase activity increased for three weeks following human lipoprotein lipase retroviral infection compared to mCherry infected mice. Human lipoprotein lipase infected mice had significantly lower blood triglycerides compared to mCherry controls and were comparable to wild-type blood triglyceride levels. Thus, targeted delivery of human lipoprotein lipase into striated muscle tissue identifies a potential therapeutic target for lipoprotein lipase deficiency.

## Introduction

Lipoprotein lipase (LPL) is primarily responsible for the breakdown of circulating triglycerides (TG) found in very low density lipoproteins and chylomicrons [1,2]. LPL is synthesized in the parenchymal cells of many tissues including adipose and striated muscle tissue. Once produced LPL is then secreted and bound by glycosylphosphatidylinositol anchored high density lipoprotein binding protein 1 (GPIHBP1) on the endothelial lumen of blood vessels. LPL then hydrolyzes circulating triglycerides into free fatty acids (FFA) for energy utilization or storage in adipose and muscle cells [3–6]. In addition, LPL aids the uptake of lipophilic vitamins and internalizes cellular lipid ligands [7–12].

Lipoprotein lipase deficiency is a rare autosomal deficiency that affects about 1 in 1 million individuals worldwide [1]. Deficiencies in LPL gene expression and/or function leads to variable hypertriglyceridemia in affected patients. In patients homozygous for LPL deficiency or compound heterozygous mutations affecting the active enzyme, high triglyceride levels are associated with markedly decreased levels of HDL cholesterol, eruptive xanthomas, lipemia retinalis, enlarged spleen and/or enlarged liver, and acute and/or chronic pancreatitis [1,13]. The absence of bioactive LPL in human patients is treated with a severely fat restricted diet or, in a small number of patients by LPL gene therapy using alipogene tiparvovec (S447X variant). Despite tiparvovec reducing the frequency of acute pancreatitis [14,15], it was recently announced that the product will not be renewed for the treatment of LPL deficiency in Europe. Importantly, despite reducing acute pancreatitis, fasting hypertriglyceridemia improved only transiently and dietary fat restriction remained necessary. Therefore, there continues to be an unmet need for new therapeutic approaches to treat both absolute and acquired LPL deficiency [16].

A mouse LPL knockout is neonatal lethal, likely arising from a buildup of triglycerides in the bloodstream leading to insufficient gas exchange in the lungs [17–20]. Transgenic expression of LPL in the liver or skeletal and cardiac muscle of LPL knockout mice rescues the neonatal lethality [21–23], but does not provide a feasible model for treating human LPL deficiency. Infection of skeletal muscle with an adeno-associated virus encoding a gain of function human LPL (hLPL) variant also rescues the neonatal lethality but is transient, as these viruses do not integrate into the genome and hLPL expression decreases over time. However, these data suggest that a permanent skeletal muscle targeted gene therapy could prove efficacious [18]. To test whether a skeletal muscle targeted gene therapy can rescue LPL deficiency, a mouse model that more closely mimics the human phenotype with hypertriglyceridemia and decreased LPL blood activity is needed. Previous attempts have generated skeletal or cardiac muscle-specific knockouts of LPL but they do not mimic the human phenotypes. A skeletal muscle-specific knockout increases insulin sensitivity in muscle, causing obesity and systemic insulin resistance, but does not cause hypertriglyceridemia [16,24]. Cardiac muscle-specific knockouts of LPL induce hypertriglyceridemia, but had no effect on LPL activity measured in post-heparin plasma [25]. Therefore, we reasoned that a skeletal and cardiac muscle-specific knockout of LPL would provide a model that more closely mimics human LPL deficiency and allow us to test a muscle-specific gene therapy.

## Results

### Mice lacking striated muscle lipoprotein lipase develop hypertriglyceridemia

**Fig 1:**
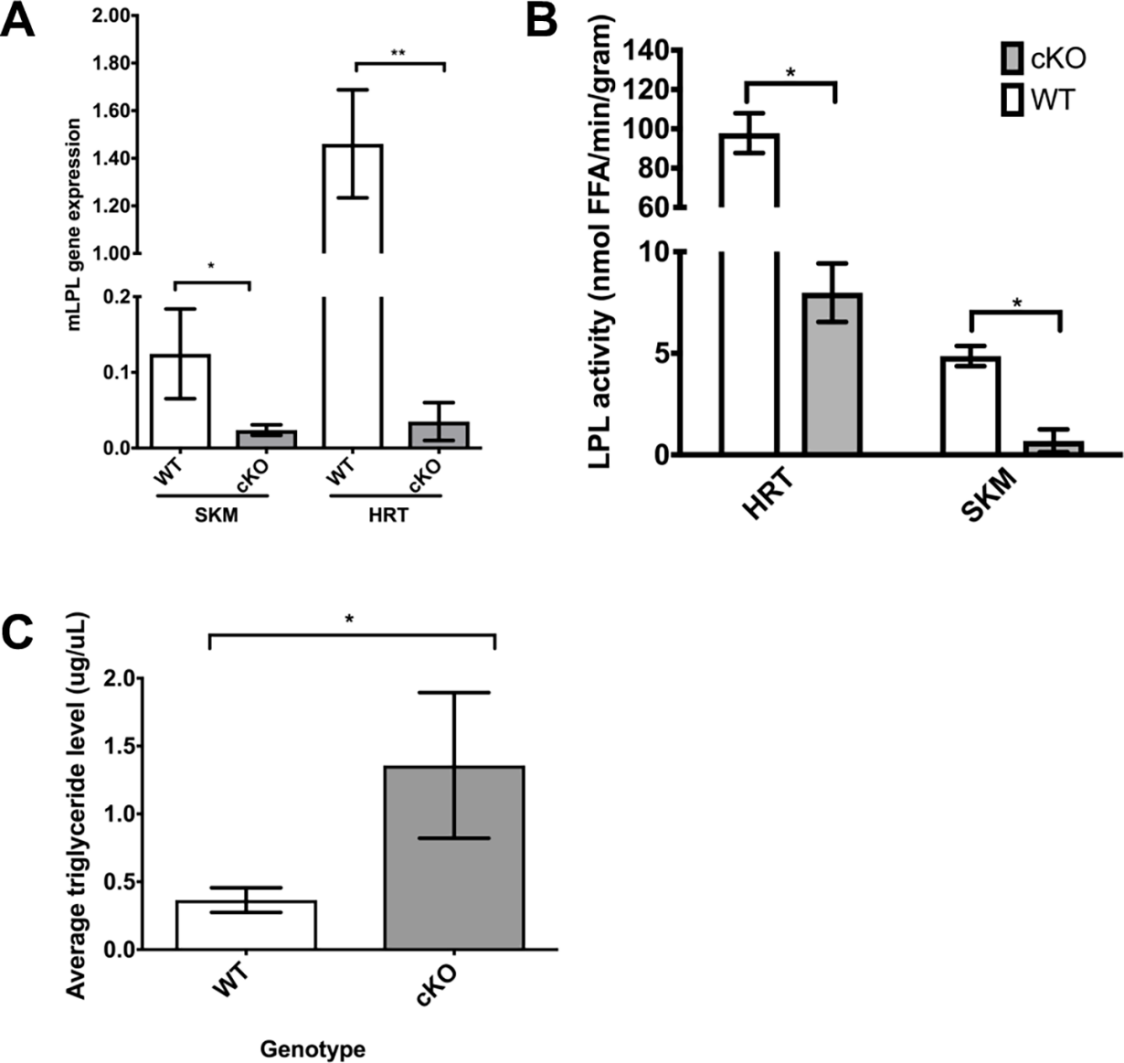
Striated muscle specific deletion of LPL leads to hypertriglyceridemia. A. Quantitative PCR for mouse LPL (mLPL) mRNA expression in skeletal muscle (SKM) or heart (HRT) from wild-type (WT) and *mck-Cre;lpl* ^*-/-*^ (cKO) mice. Mouse LPL is virtually undetectable in cKO skeletal and cardiac muscles compared to WT tissue (*p<0.02, **p<0.001; student’s T-test). Error bars represent standard deviation (SD). N = 3 for each genotype. B. Lipoprotein lipase enzyme activity in heart (HRT) and skeletal muscle (SKM) from wild-type (WT) and *mck-Cre;lpl* ^*-/-*^ (cKO) mice. LPL enzyme activity is significantly decreased in cKO samples compared to WT (p<0.02; Student’s t-test). Error bars represent SD. FFA = free fatty acids. N = 3 for each genotype. C. Average fasting serum triglyceride levels from wild-type (WT) and *mck-Cre;lpl* ^*-/-*^ (cKO) mice. cKO mice have significantly higher fasting triglycerides levels than WT mice (p<0.0001, Student’s t-test). Error bars represent SD. N = 3 for each genotype.

To develop a tractable model to test rescuing LPL deficiencies in adult mice we asked if knocking out LPL in differentiated striated (skeletal and cardiac) muscle using a muscle creatine kinase (mck) Cre would cause both hypertriglyceridemia and lower post-heparin plasma LPL activity. Mice homozygous (*mck-Cre;lpl*^*-/-*^*)* for the floxed LPL gene (LPL cKO) or heterozygous for the floxed LPL gene were bred, exhibited normal Mendelian ratios at birth and appeared healthy (data not shown). In six-month-old mice, LPL mRNA was nearly undetectable in either skeletal muscle tissue or cardiac tissue from LPL cKO mice compared to wild type mice (Fig 1A, p<0.02). Consistent with the low level of LPL mRNA, tissue specific LPL activity in LPL cKO mice compared to wild type was 10-fold lower in skeletal muscle and 50-fold lower in cardiac muscle than in wild type (Fig 1B, p<0.02). The reduction in LPL expression and LPL activity increased fasting serum triglyceride levels in LPL cKO mice compared to wild-type mice (Fig 1C, p<0.0001).

### hLPL Production by RCAS-LPL Infection

**Fig 2:**
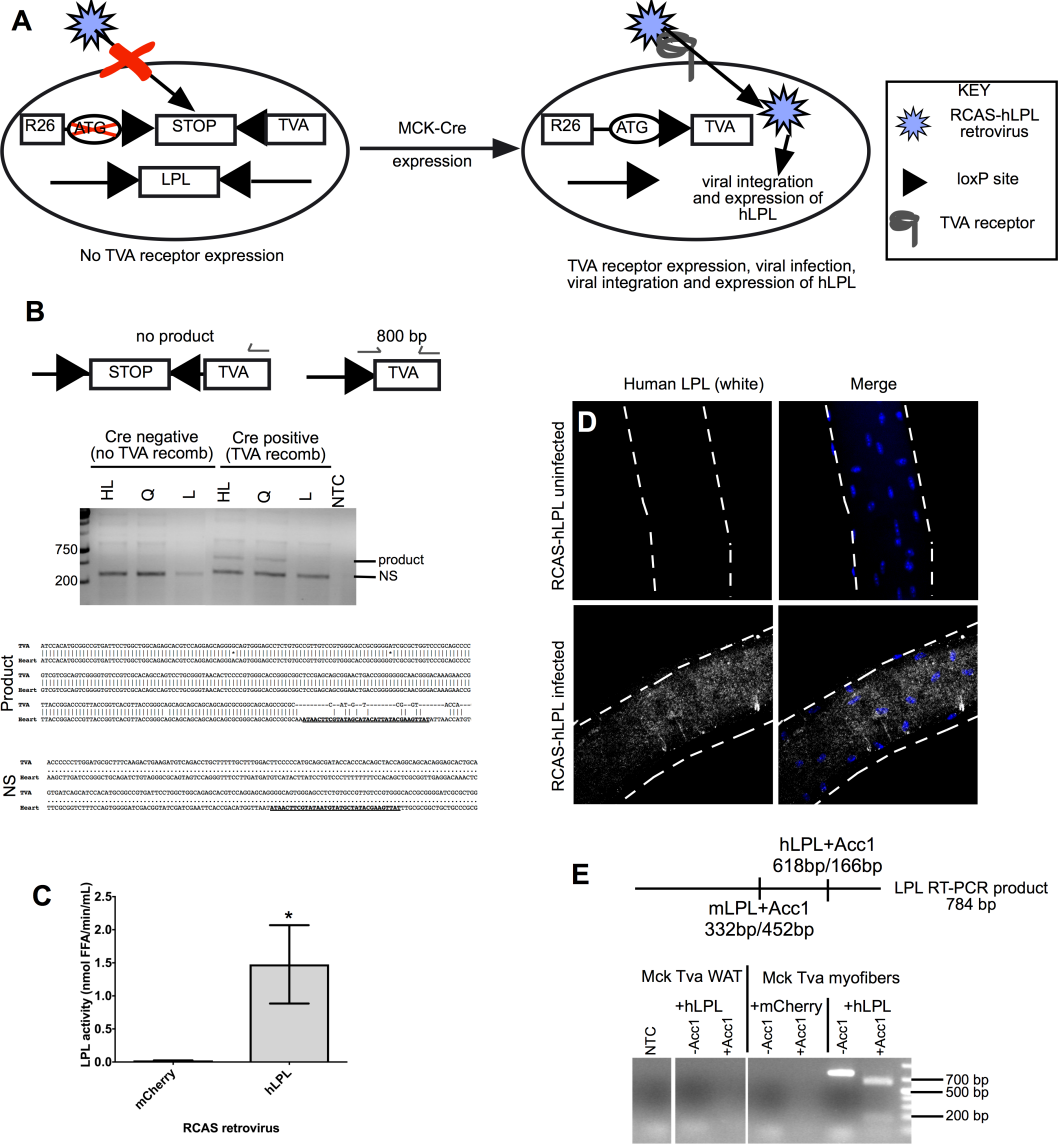
RCAS retroviral infection, integration and expression *in vitro*. A. An illustration of Mck-Cre-mediated recombination of the Rosa26 locus and LPL conditional allele in *mck-Cre;lpl* ^*-/-*^*;Rosa26*^*LSLTVA* lacZ+/-^ mice. Cre-mediated recombination deletes LPL and enables Tva expression in striated muscle. Tva expression is required for RCAS retroviral infection, integration and expression in striated muscle. B. (Upper) A diagram of PCR primer binding sites in *Rosa26*^*LSLTVA lacZ+/-*^ allele for assessing Cre-mediated recombination efficiency. (Middle) An 800 bp PCR product is present in muscles of *mck-Cre;Rosa26*^*LSLTVA* lacZ+/-^ mice but not in liver tissue. The 800 bp PCR product is detectable in hindlimb (HL) and quadriceps (Q) of *mck-Cre; Rosa26*^*LSLTVA* lacZ+/-^ mice. A PCR product is not detectable in in liver (L) demonstrating specificity of Cre-mediated recombination. NS denotes a non-specific product at 400 bp. NTC denotes non-template control. (Lower) Representative sequencing alignments of the TVA gene and the sequencing product as well as the non-specific product (NS) obtained from heart gDNA PCR products excised from an agarose gel. LoxP sequences are bold and underlined in each sample. N = 3 for each condition; representative gel image and sequencing alignment shown. C. Active LPL enzyme is secreted from chicken fibroblast cells (DF1) transfected with RCAS plasmid encoding hLPL. No LPL enzyme activity is detected in media from DF1 cells transfected with RCAS plasmid encoding mCherry (p<0.003; Student’s t-test). Error bars represent SD. FFA = free fatty acids. N = 3 for each condition. D. Immunofluorescence images of myofibers isolated from *mck-Cre;lpl* ^*-/-*^*;Rosa26*^*LSLTVAlacZ+/-*^ mice 72 hours post-infection *in vitro* with RCAS-hLPL and immunostained for human LPL using an anti-human-specific LPL antibody, 5D2. Human LPL immunoreactivity is present in the myofiber infected with RCAS-hLPL and not detectable in the uninfected control myofiber. Nuclei are counterstained with DAPI (blue) and myofibers are outlined with dotted lines. E. (Upper) A diagram of the restriction fragment length polymorphism used to distinguish between human and mouse LPL mRNA expression in RCAS-hLPL infected myofibers. The Acc1 enzyme digests both the human and mouse RT-PCR products generating unique distinguishable products for each species. (Lower) A ~780 bp band showing amplification of LPL only in myofibers infected with RCAS-hLPL (-Acc1). After digestion (+Acc1) the LPL RT-PCR product is digested into two distinct bands at ~600 bp and ~200 bp, indicative of hLPL not mLPL. No hLPL mRNA expression is detected in myofibers infected with RCAS-mCherry or in white adipose tissue (WAT) from mice infected with RCAS-hLPL retrovirus. NTC denotes non-template control. N = 3 for all conditions; a representative gel image shown.

To rescue LPL loss in skeletal and cardiac muscle, we chose the RCAS (Replication Competent Avian Sarcoma-leukosis virus long-terminal repeat with splice acceptor) avian retrovirus to deliver the human LPL (hLPL) gene. RCAS(A) requires the Tva (Tumor virus A) receptor on the cell surface of for retroviral infection and can infect both dividing and non-dividing cells [26]. To generate a mouse that lacks LPL and expresses Tva in terminally differentiated striated muscle, we crossed *mck-Cre;lpl*^*-/-*^ mice with a ROSA26 lox-stop-lox (LSL) Tva mouse [27]. The resultant mouse (*mck-Cre;lpl*^*-/-*^*;Rosa26*^LSL*TVA(lacZ)+/-*^*)* simultaneously expresses Tva in striated muscle as well as knocking out LPL in striated muscle, permitting RCAS retroviral infection, integration, and ectopic gene or shRNA expression only in terminally differentiated striated muscle (Fig 2A) [28].

To assess recombination efficiency of the ROSA locus in *mck-Cre;lpl*^*-/-*^*; Rosa26*^LSL*TVA(lacZ)+/-*^ mice (LPL cKO Tva) we performed PCR on skeletal muscle gDNA with primers specific for the recombined *Rosa26*^LSL*TVA(lacZ)+/-*^ allele, confirming a PCR product in skeletal muscle but not in tissues that do not express MCK (Fig 2B). We verified recombination by sequencing both the recombination product and the non-specific amplification product. The recombination product contained one loxP sequence TVA sequence, indicating removal of the stop codon, whereas the non-specific product did not contain either loxP or TVA sequences (Fig 2B). The non-specific products in liver and other non MCK expressing tissues also did not align to the TVA gene. Replication-competent RCAS viruses accommodate between 2 and 2.5 kb of ectopic DNA, permitting insertion of the entire 1.43 kb sequence encoding hLPL. Active LPL is present in media harvested from chicken DF1 cells, one week following transfection with RCAS-hLPL but was not present in media of RCAS-mCherry transfected DF-1 cells (Fig 2C; p<0.003), demonstrating that hLPL protein is produced, secreted, and active, when transcribed from the retroviral LTR. Since we planned to express LPL by infection of differentiated striated muscle tissue, we assessed whether single muscle fibers (myofibers) isolated from LPL cKO Tva mice were competent for RCAS infection, integration, and expression of hLPL. Myofibers from LPL cKO Tva mice were isolated, infected with RCAS-hLPL virus, cultured for 72 hours, and assessed for hLPL mRNA and protein expression (Fig 2D and 2E). Human LPL mRNA and protein were detected in infected myofibers indicating successful RCAS-hLPL infection, integration and hLPL production., Human LPL mRNA was not detected in myofibers infected with RCAS-mCherry virus or in white adipose tissue, neither of which express TVA.

### *In vivo* infection with RCAS retrovirus produces hLPL

**Fig 3:**
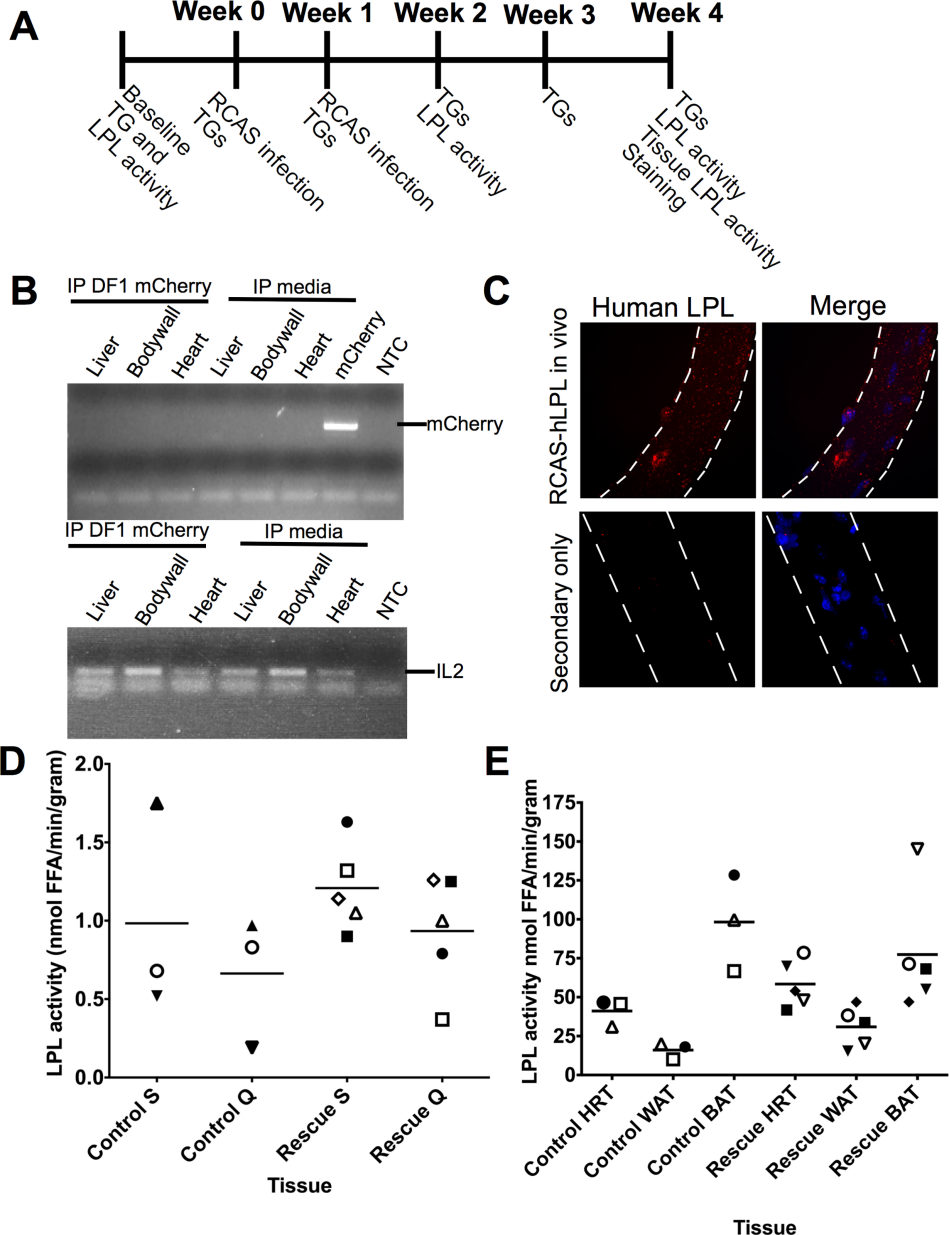
Expression of hLPL in striated muscle following *in vivo* infection with RCAS-hLPL. A. A schematic of the RCAS rescue strategy and data collection time points. RCAS-hLPL or RCAS-mCherry producing cells were injected into mice at week 0 and 1. Fasting serum triglycerides (TG) were measured at all indicated time points. Post-heparin LPL activity (LPL activity) was measured at baseline, week 2 and week 4 post infection. At 4 weeks post infection, animals were sacrificed, striated muscle samples analyzed for tissue specific LPL activity and myofibers isolated to perform immunostaining for hLPL. B. (Upper) Representative PCR for mCherry gDNA from various tissues of *mck-Cre;lpl*^*-/+*^*;Rosa26*^*LSLTVA lacZ+/+*^ mice two weeks post-injection with RCAS-mCherry DF1 cells or media control. No amplification of mCherry was detected in any tissue two weeks post injection. mCherry plasmid was used as a positive control for amplification. (Lower) Interleukin-2 (IL2) gDNA amplification as a control for gDNA input into original mCherry reaction. NTC = non-template control. n = 3 for each condition. C. Immunofluorescence images of myofibers isolated from *mck-Cre;lpl*^*-/-*^*;Rosa26*^*LSLTVA lacZ+/-*^ four weeks post injection with DF1 cells producing RCAS-hLPL retrovirus. Human LPL immunoreactivity is present throughout the myofiber isolated from RCAS-hLPL infected mice and absent from uninjected mice. Nuclei are counterstained with DAPI (blue) and myofibers are outlined with dotted lines. D. Tissue-specific LPL activity in soleus muscle (S) or quadriceps muscle (Q) from *mck-Cre;lpl*^*-/-*^*;Rosa26*^*LSLTVA lacZ+/-*^ mice four weeks after injection with RCAS-hLPL DF1 cells (rescue; n = 5) or four weeks post-injection of RCAS-mCherry DF1 cells (control; n = 3). Each symbol represents an individual mouse and bars represent the mean of all samples. E. Tissue specific LPL activity in heart muscle (HRT), white adipose tissue (WAT), or brown adipose tissue (BAT) from *mck-Cre;lpl*^*-/-*^*;Rosa26*^*LSLTVA lacZ+/-*^ mice four weeks after injection with RCAS-hLPL DF1 cells (rescue; n = 5) or four weeks after injection with RCAS-mCherry DF1 cells (control; n = 3). WAT and BAT served as positive controls as these tissues produce LPL but do not express MCK. Each symbol represents an individual mouse and bars represent the mean of all samples.

Infection of LPL cKO Tva mice with RCAS-LPL retroviruses by tail vein injection yielded no detectable hLPL immunoreactivity in isolated myofibers or significant changes in serum triglyceride levels 2 weeks post-infection (data not shown). To increase delivery of the retrovirus, we injected DF1 cells producing either RCAS-mCherry or RCAS-hLPL retrovirus twice i.p., with a second injection one week following the first injection (Fig 3A). To confirm that DF1 cells do not remain in mice following injection, we injected DF1 cells producing RCAS-mCherry into wild-type mice. Two weeks later gDNA was isolated from tissues within the body cavity and we performed PCR for mCherry gDNA. No mice injected with RCAS-mCherry producing DF1 cells had detectable mCherry gDNA indicating that DF1 cells are cleared from the body within two weeks of injection (Fig 3B). Four weeks following the initial injection, skeletal muscle was assessed for hLPL production. Intact myofibers isolated from DF1 cell injected mice were isolated from the EDL muscles four weeks post-injection. Myofibers isolated from mice injected with DF1 cells producing RCAS-hLPL virus were immunoreactive for hLPL (Fig 3C) demonstrating that virus produced by DF1 cells infects striated muscle and the infected muscle produces and accumulates hLPL protein. Uninfected mice or mice infected with DF1 cells producing RCAS-mCherry (data not shown) are not immunoreactive for hLPL.

One month following the initial injection of cells secreting RCAS-hLPL or RCAS-mCherry retrovirus, we assessed the levels of LPL activity in the quadriceps, soleus, and heart muscles. Brown and white adipose tissues produce varying levels of LPL, serving as a positive controls [2,29]. Additionally, neither brown adipose tissue nor white adipose tissue can be infected by RCAS as these tissues do not express MCK [26]. Tissue-specific LPL activity was elevated in skeletal and cardiac muscle from mice injected with RCAS-hLPL DF1 cells compared to mice injected with RCAS-mCherry DF1 cells (Fig 3D). However, due to the variability of LPL activity in individual mice, the means were not significantly different between the tissues from the LPL injected and mCherry injected mice (Fig 3D). LPL activity was elevated in brown adipose tissue as compared to white adipose tissue and was unchanged in adipose tissue from RCAS-LPL infected mice compared to adipose tissue from RCAS-mCherry infected mice (Fig 3E). Thus, hLPL released by striated muscle does not appear to accumulate in adipose tissue and instead may be trafficked to blood vessels.

### Infection with RCAS-hLPL retrovirus rescues hypertriglyceridemia

**Fig 4:**
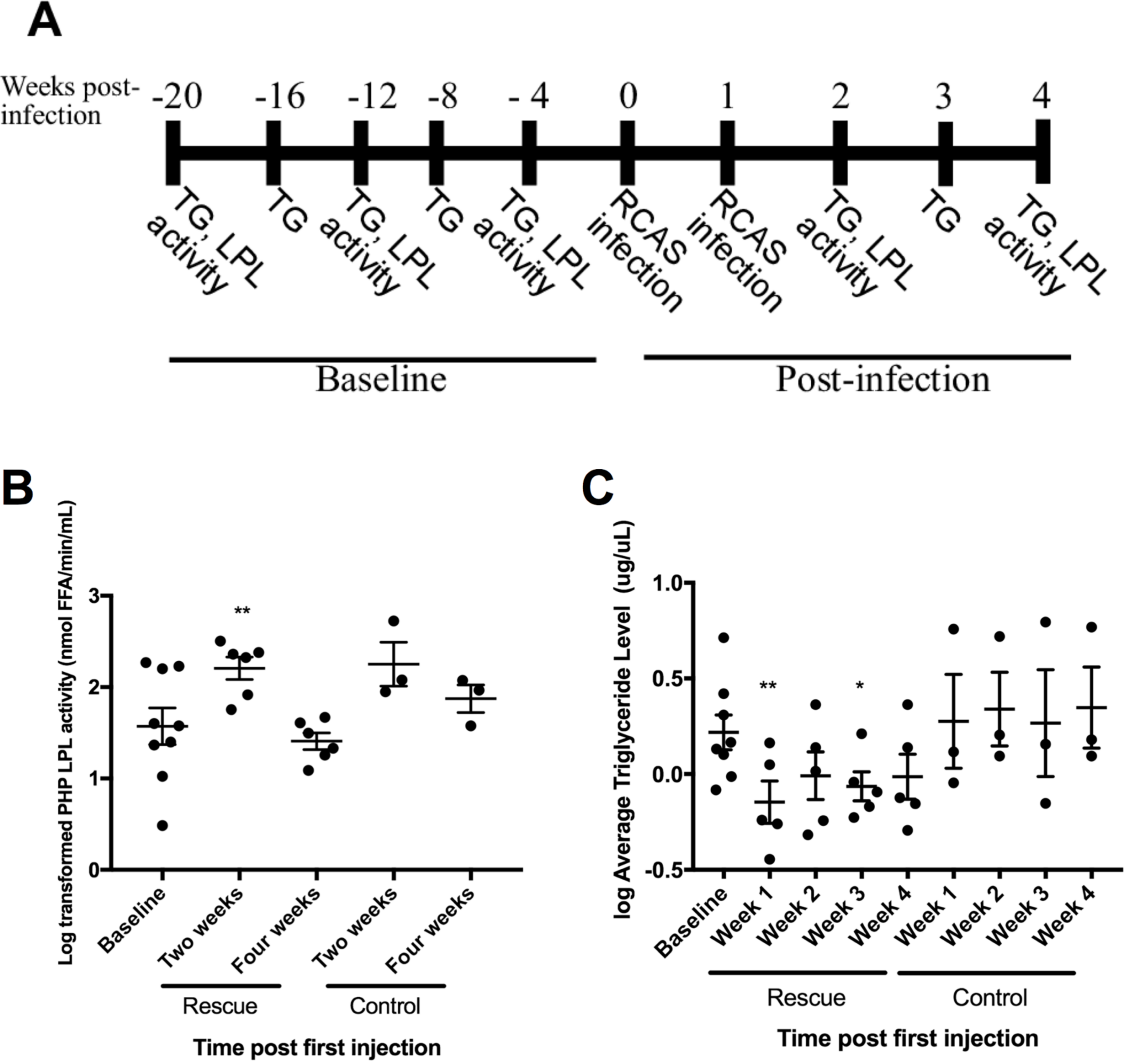
*In vivo* RCAS-hLPL infection decreases high triglyceride levels in hypertriglyceridemic mice. A. A schematic showing the timing measurements for fasting serum triglycerides (TG) or post-heparin LPL activity (PHP LPL) taken prior to (baseline) and following (post-infection) injection of RCAS-hLPL or RCAS-mCherry producing cells. Fasting serum triglycerides (TG) were measured at all indicated time points. B. PHP LPL activity was measured at baseline, week 2 and week 4 post infection. Two weeks post-injection, rescue animal PHP LPL activity is significantly higher than at baseline (p<0.02; One-way ANOVA performed on log transformed values). PHP LPL activity beings to decline by one month post-infection, but remains higher than baseline. PHP LPL activity levels were normalized to baseline levels. Graph shows log transformed values, bars represent the mean of all samples, error bars show +/- SEM. C. Average log transformed fasting serum triglyceride levels from *mck-Cre;lpl*^*-/-*^*;Rosa26*^*LSLTVA lacZ+/-*^ mice prior (baseline) and weekly post-injection with RCAS-hLPL DF1 cells (rescue; n = 5) or post-injection with RCAS-mCherry DF1 cells (control; n = 3). At one and three weeks post-infection, rescue mice have significantly lower triglyceride levels compared to baseline (* p<0.05, **p<0.02, One-way ANOVA). By four weeks post injection, rescue triglyceride levels begin to rise, but remain lower than baseline. Triglyceride levels in control mice do not change significantly from baseline following injection of RCAS-mCherry producing cells. Graph shows log transformed values, bars represent the mean of all samples, error bars show +/- SEM.

We conducted longitudinal assays for endothelial lumen-associated LPL and triglyceride levels to determine if infection with RCAS-hLPL increased post-heparin plasma LPL activity and reduced serum triglyceride levels. Serum triglyceride levels and post-heparin plasma LPL activity assays were conducted at 4 week intervals beginning 20 weeks prior to infection. Serum triglyceride levels were measured weekly post-injection and post-heparin plasma LPL activity was measured again at 2 weeks and 4 weeks post-injection (Fig 4A). Two weeks post-injection post-heparin plasma LPL activity was significantly higher in mice injected with RCAS-hLPL producing cells compared to mice injected with RCAS-mCherry producing cells (Fig 4B). One month following the initial RCAS-hLPL cell injection, post-heparin plasma LPL activity decreased but remained higher than baseline and controls (Fig 4B; p<0.02). The increases in post-heparin plasma LPL activity following RCAS-hLPL injection demonstrated that functional hLPL was produced by striated muscle and is appropriately trafficked to the endothelial lumen, suggesting that plasma triglyceride levels may differ in rescue and control mice. We longitudinally measured fasting plasma triglyceride levels prior to injection to determine baseline values for all mice (Fig 4C; baseline). Fasting plasma triglycerides were then assayed at weekly intervals following injection of RCAS-hLPL or RCAS-mCherry producing cells. RCAS-hLPL infected LPL cKO Tva mice had significantly lower fasting plasma triglyceride levels at one and three weeks post injection compared to baseline (Fig 4C; p<0.03). We observed no change in the fasting serum triglyceride levels of LPL cKO Tva mice infected with RCAS-mCherry, indicating an injection of RCAS producing cells itself did not alter serum triglyceride levels in LPL cKO Tva mice.

## Discussion

Lipoprotein lipase deficiency is a rare, orphan disease that is managed with a severely fat restricted diet. Patients have received some metabolic benefits with alipogene tiparvovec, however it appears that this therapy will soon no longer be available [14,15]. Previously generated mouse models for LPL deficiency are unable to mimic both the hypertriglyceridemia and decreased plasma LPL activity found in human patients [16,25]. Thus, we developed a new mouse model for LPL deficiency that exhibits hypertriglyceridemia and significantly reduced striated muscle LPL activity by employing a striated muscle specific Cre driver to conditionally knockout striated muscle LPL. Removal of LPL by Cre during skeletal and cardiac muscle development does not mimic LPL deficiencies in human patients, however, the mouse provides an improved system than those previously available for testing alternative gene therapies to reduce hypertriglyceridemia associated with LPL deficiency.

Mice null for LPL die shortly after birth unless rescued by adeno-associated viral delivery to the liver or skeletal muscle [21,23]. Although a one-time intramuscular administration of the AAV vector alipogene tiparvovec in patients with LPL deficiency reduced plasma triglycerides by ~50% they returned to baseline ~ 6 months later [14]. Nevertheless, patients did experience persistent LPL activity, sustained improvements in postprandial chylomicron metabolism and decreased incidence of pancreatitis one year following infection. To test an alternative method for long-term expression of LPL from skeletal muscle, we employed retroviral infection where the retrovirus is targeted only to striated muscle by conditional expression of Tva, an avian retroviral receptor. Initially, retroviral delivery was attempted by tail vein injections of RCAS-hLPL but upon observing no changes in LPL activity or serum triglyceride levels we opted instead to inject avian cells producing retrovirus for sustained viral production. Two weeks post injection of RCAS-hLPL producing cells, LPL activity was detectable in post-heparin plasma and by three weeks post injection, triglyceride levels were reduced in mice infected with RCAS-hLPL retrovirus compared to controls. While LPL activity in muscles isolated from RCAS-hLPL cell injected mice was elevated, the levels were not significantly different from skeletal muscle LPL activity in RCAS-mCherry cell injected mice, despite a significant increase in post heparin LPL activity in RCAS-hLPL injected mice. It is possible that low levels of hLPL produced by various striated muscles accumulate and are sufficient to elevate LPL activity in the plasma reducing triglyceride levels. At one month following infection heparin-releasable LPL activity decreased significantly, suggesting either a reduction of hLPL production *in vivo* or the mice developed antibodies against the human LPL protein. Despite the reduction in post-heparin LPL activity, triglyceride levels remained low one month post-infection, demonstrating that sufficient hLPL was produced systemically to reduce triglycerides. Thus, production of LPL by striated muscle reversed hypertriglyceridemia arising from knock out of striated muscle LPL. Retroviral delivery of hLPL is not a viable treatment for human hypertriglyceridemia, but these experiments demonstrate that long-term expression of hLPL in skeletal muscle will likely reduce or even eliminate hypertriglyceridemia wherein total absence of LPL is not present. As alternatives to retroviral delivery, transduction of hLPL into skeletal muscle could be accomplished by AAV-mediated transduction, or by engineering skeletal muscle stem cells to express hLPL. Upon fusion of satellite cells into myofibers during muscle maintenance [30], permanent hLPL production from skeletal muscle will ensue and may be sufficient to reduce hypertriglyceridemia in human patients, providing an alternative to viral-mediated gene therapy.

## Materials and methods

### Mice

Mice were bred and housed at the University of Colorado Boulder or University of Colorado Denver Anschutz Medical Campus in a pathogen-free facility per the National Institute of Health (NIH) guidelines for ethical treatment of animals. Rescue experiments were performed on male *mck-Cre;lpl*^*-/-*^*;Rosa26*^*LSLTVA(lacZ) +/-*^ mice that were 6–8 months old at the time of infection. All procedures involving removal of tissues were performed on euthanized animals. Animals were euthanized with compressed carbon dioxide, an accepted method by the Panel on Euthanasia of the American Veterinary Medical Association. For blood draws mice were anesthetized with isoflurane gas provided in a manifold. For tail vein injections mice were placed in a restraint. Retroviral injections were delivered via i.p. with a 25g needle. Mice were monitored for fifteen minutes following injection of retrovirus or blood draws then hourly for 4 hours to insure no signs of distress. All protocols were approved by the IACUC at the University of Colorado Boulder or University of Colorado Denver Anschutz Medical Campus.

### Quantitative PCR

RNA was extracted from skeletal and cardiac muscle from 6 month old LPL cKO or wild-type control mice using the RNeasy kit (Qiagen, Germantown, PA) according to manufacturer instructions. Total RNA was reverse transcribed into cDNA with iScript cDNA synthesis kit (Bio-Rad, Hercules, CA) and quantitative PCR performed using primers sets for LPL and two reference genes with iTaq Universal SYBR Green Supermix (Bio-Rad, Hercules, CA) per manufacturer’s instructions. Reactions were run in triplicate on an iQ5 Real Time PCR instrument (Bio-Rad, Hercules, CA). Data were normalized using the comparative Ct method to two reference genes. Primer sequences available on request. Statistics and analysis were performed in Prism (GraphPad, La Jolla, CA).

### PCR for LSL Tva allele recombination

Genomic DNA was isolated from hind limb, heart and liver tissue with a Qiagen DNeasy kit (Qiagen, Germantown, PA) according to manufacturer instructions. Standard Taq polymerase (New England Biosciences, Ipswich, MA) was used according to manufacturer instructions using primers targeting the Rosa26 locus following removal of the lox-stop-lox cassette. ROSA26 lox-stop-lox Tva recombination primers were a gift from D. Sauer. Individual bands were isolated from a 1.5% agarose gel (Qiagen, Germantown, PA) according to manufacturer instructions and Sanger sequenced to confirm LSL recombination in MCK expressing tissues.

### RCAS virus generation and infection

RCASBP(A) vectors containing Gateway cloning sites were ordered from Addgene. Human lipoprotein lipase (rescue) or mCherry (control) genes were subcloned into the RCASBP(A) vector using attb recombination sites. RCAS retroviruses were generated in DF-1 chicken fibroblast cells with standard protocols concentrated and stored at -80°C until use for *in vitro* studies. For *in vivo* rescue studies 1x10^6^ DF1 cells producing RCAS-hLPL or RCAS-mCherry retrovirus were injected into *mck-Cre;lpl*^*-/-*^*;Rosa26*^*Tva(lacZ) +/-*^ recipient mice via intraperitoneal injection (once weekly for two weeks) to infect differentiated striated muscle. Weekly fasting serum triglyceride levels and biweekly post-heparin blood LPL activity levels were examined following injection as described below. One month following initial infection mice were sacrificed and tissue examined for LPL activity and immunofluorescent staining for hLPL protein expression.

### Isolation, Infection, RT-PCR-RFLP and Immunofluorescent Staining of Myofibers

Individual myofibers were isolated from hind limb muscles and cultured as described previously [31]. For *in vitro* infection studies approximately 200 myofibers were incubated with 3x10^6^ RCAS-hLPL or RCAS-mCherry retrovirus for 2 hours to allow infection. Myofibers were then washed with F12-C+15% horse serum and cultured for 72 hours. After 72 hours in culture myofibers were either collected for RNA or fixed for immunostaining. RNA was collected from 200 myofibers using a RNeasy kit (Qiagen, Germantown, PA) and reverse transcribed into cDNA using Superscript III (Life Technologies, Carlsbad, CA) according to manufacturer’s instructions. Human and mouse LPL mRNA was amplified using Phusion Taq (New England Biosciences, Ipswich, MA) and the following primers: forward 5’ AGCAAAGCCCTGCTCGTCGTGACT and reverse 5’ GGTCCACATCTCCAAGTCC. A restriction digest was performed on the RT-PCR reaction following amplification of LPL using Acc1 (New England Biosciences, Ipswich, MA) according to manufacturer’s instructions. Myofibers that were fixed for immunostaining were blocked as described previously [31] and incubated with 4 μg/mL mouse anti-human lipoprotein lipase antibody (5D2) for one hour at room temperature. Myofibers were then washed, stained with anti-mouse AlexFlour 555 (Life Technologies, Carlsbad, CA), DAPI, dry mounted on slides and set with MOWIOL [31]. All immunofluorescent images were taken on a Nikon spinning disc confocal microscope housed in the MCDB Core Microscopy facility at the University of Colorado Boulder.

### Lipoprotein lipase and triglyceride assays

Baseline fasting serum triglyceride levels and post-heparin LPL activity levels were collected monthly or bimonthly, respectively, for 5 months prior to rescue. For fasting serum triglyceride levels mice were fasted 4 hour prior to blood collection. Serum triglyceride levels were performed as previously described [16]. Samples for post-heparin blood LPL activity were collected from fasting animals prior to and 15 minutes following tail vein injections of heparin (100 units/kg) and LPL activity measured as previously described [32]. Tissue specific LPL activity assays were performed on isolated striated muscle groups (soleus, quad, heart), white and brown adipose tissue following homogenization and LPL release with heparin. Once released from the tissue, LPL activity was performed as described for blood samples [32]. Statistics and analysis were performed in Prism (GraphPad, La Jolla, CA).
